# All-*trans* retinoic acid alleviates transmissible gastroenteritis virus-induced intestinal inflammation and barrier dysfunction in weaned piglets

**DOI:** 10.1186/s40104-023-00978-2

**Published:** 2024-02-09

**Authors:** Junning Pu, Daiwen Chen, Gang Tian, Jun He, Ping Zheng, Zhiqing Huang, Xiangbing Mao, Jie Yu, Yuheng Luo, Junqiu Luo, Hui Yan, Aimin Wu, Bing Yu

**Affiliations:** 1 Institute of Animal Nutrition, Sichuan Agricultural University, Chengdu, 611130 Sichuan People’s Republic of China; 2 Key Laboratory of Animal Disease-Resistance Nutrition, Ministry of Education, Ministry of Agriculture and Rural Affairs, Key Laboratory of Sichuan Province, 611130 Chengdu, Sichuan People’s Republic of China

**Keywords:** All-*trans* retinoic acid, Inflammation, Intestinal barrier, Piglets, Transmissible gastroenteritis virus

## Abstract

**Background:**

Transmissible gastroenteritis virus (TGEV) is one of the main pathogens causing severe diarrhea of piglets. The pathogenesis of TGEV is closely related to intestinal inflammation. All-*trans* retinoic acid (ATRA) is the main active metabolite of vitamin A, which has immunomodulatory and anti-inflammatory properties. However, it is unclear whether ATRA can alleviate TGEV-induced intestinal inflammation and barrier dysfunction in piglets. This study aimed to investigate the effects of ATRA on growth performance, diarrhea, intestinal inflammation and intestinal barrier integrity of TGEV-challenged piglets.

**Methods:**

In a 19-d study, 32 weaned piglets were randomly divided into 4 treatments: Control group (basal diet), TGEV group (basal diet + TGEV challenge), TGEV + ATRA5 group (basal diet + 5 mg/d ATRA + TGEV challenge) and TGEV + ATRA15 group (basal diet + 15 mg/d ATRA + TGEV challenge). On d 14, piglets were orally administered TGEV or the sterile medium.

**Results:**

Feeding piglets with 5 and 15 mg/d ATRA alleviated the growth inhibition and diarrhea induced by TGEV (*P* < 0.05). Feeding piglets with 5 and 15 mg/d ATRA also inhibited the increase of serum diamine oxidase (DAO) activity and the decrease of occludin and claudin-1 protein levels in jejunal mucosa induced by TGEV, and maintained intestinal barrier integrity (*P* < 0.05). Meanwhile, 5 mg/d ATRA feeding increased the sucrase activity and the expressions of nutrient transporter related genes (*GLUT2* and *SLC7A1*) in jejunal mucosa of TGEV-challenged piglets (*P* < 0.05). Furthermore, 5 mg/d ATRA feeding attenuated TGEV-induced intestinal inflammatory response by inhibiting the release of interleukin (IL)-1β, IL-8 and tumor necrosis factor-α (TNF-α), and promoting the secretion of IL-10 and secretory immunoglobulin A (sIgA) (*P* < 0.05). Feeding 5 mg/d ATRA also down-regulated the expressions of Toll-like receptors and RIG-I like receptors signaling pathway related genes *(TLR3*, *TLR4*, *RIG-I*, *MyD88*, *TRIF* and *MAVS*) and the phosphorylation level of nuclear factor-κB-p65 (NF-κB p65), and up-regulated the inhibitor kappa B alpha (IκBα) protein level in jejunal mucosa of TGEV-challenged piglets (*P* < 0.05).

**Conclusions:**

ATRA alleviated TGEV-induced intestinal barrier damage by inhibiting inflammatory response, thus improving the growth performance and inhibiting diarrhea of piglets. The mechanism was associated with the inhibition of NF-κB signaling pathway mediated by TLR3, TLR4 and RIG-I.

**Graphical Abstract:**

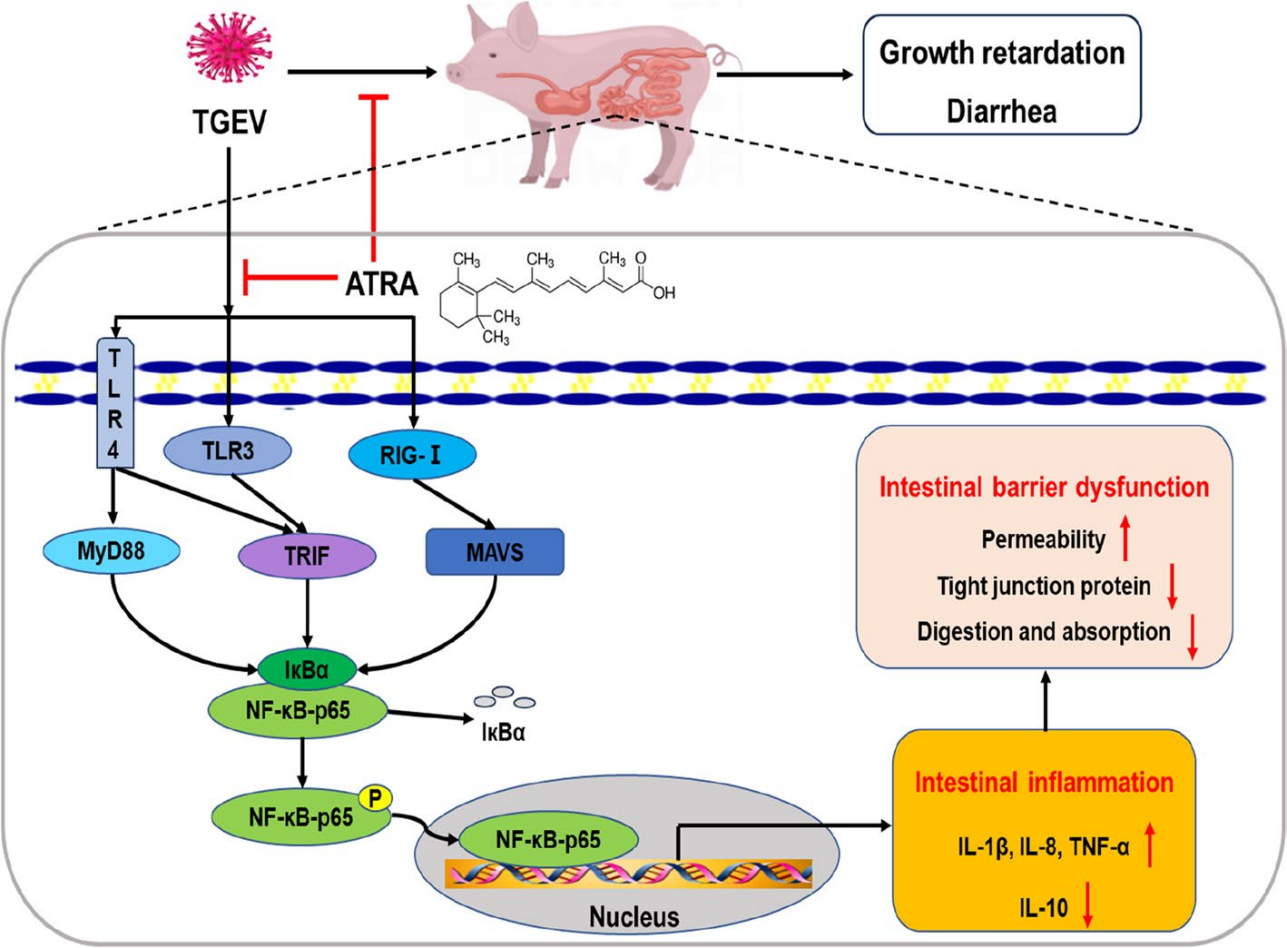

## Background

Transmissible gastroenteritis (TGE) caused by transmissible gastroenteritis virus (TGEV) infection is an acute and highly contagious infectious intestinal disease, which is characterized by vomiting, dehydration and severe diarrhea, and causes up to 100% mortality of piglets less than 2 weeks old [1]. At present, TGE is still one of the most important diseases threatening pork production in the world, which brings significant economic losses to the pig breeding industry [2]. The small intestine is not only the major digestive and absorption organ of nutrients, but also an important barrier against the invasion of pathogenic microorganisms and toxins [3]. Previous studies have shown that the porcine small intestine is the main target organ of TGEV infection [4]. Previous studies have reported that TGEV infection can induce significant inflammation response in the gastrointestinal mucosa of piglets, which leads to the damage of intestinal barrier structure and function, and then affects the transport of nutrients, increases the osmotic pressure in the intestinal lumen, and finally leads to severe diarrhea and dehydration [5, 6]. Consistent with pathological changes in vivo, TGEV infection also induced inflammatory injury of ST cells and IPEC-J2 cells in vitro [7, 8]. This indicates that the inflammatory response plays an important role in the pathogenicity of TGEV. Therefore, suppressing intestinal inflammation may be a potential strategy to alleviate the damage of intestinal barrier structure and function induced by TGEV.

All-*trans* retinoic acid (ATRA) is the main active metabolite of vitamin A, which plays an important role in regulating immune function and anti-inflammation [9]. Previous studies have found that ATRA can up-regulate the levels of anti-inflammatory cytokines TGF-β1 and IL-10 by mediating RARα and RARβ expressions, thus alleviating the epididymitis induced by *Escherichia coli* in mice [10]. Gu et al. [11] found that ATRA can attenuate lipopolysaccharide-induced inflammatory responses by suppressing TLR4/NF-κB signaling pathway in rat mammary tissue. Furthermore, recent studies have shown that ATRA, as an immunomodulator, also plays an important role in relieving intestinal inflammation and maintaining intestinal homeostasis [12]. Previous studies have found that in the experimental mouse IBD model, pharmacological blocking of the retinoic acid degrading enzyme CYP26A1 can restore the level of ATRA in the body and reduce intestinal inflammation [13]. Mielke et al. [14] found that ATRA could inhibit colitis induced by dextran sulfate sodium (DSS) or *Citrobacter rodent* infection by promoting the production of IL-22, Reg3β and Reg3γ by γδ T cells and innate lymphoid cells in colon. Hong et al. [15] reported that ATRA attenuates DSS-induced murine colitis by suppressing NF-κB signaling pathway. In addition, our previous studies found that ATRA can attenuate TGEV-induced inflammatory injury in IPEC-J2 cells via suppressing the RLRs/NF-κB signaling pathway [16]. However, it is unclear whether ATRA can alleviate TGEV-induced intestinal inflammation and epithelial damage in weaned piglets.

Accordingly, based on the anti-inflammatory ability of ATRA, we hypothesized that ATRA could alleviate TGEV-induced intestinal barrier injury in piglets by inhibiting inflammation response, thereby inhibiting diarrhea and improving growth performance of piglets. Therefore, this study aimed to investigate the effects of ATRA on growth performance, diarrhea, intestinal inflammation and intestinal barrier integrity in TGEV-infected piglets.

## Materials and methods

### Animals, diets and experimental design

All animal procedures used in this study were approved by the Animal Care and Use Committee of Sichuan Agricultural University (license number: CD-SYXK-2017-015). In a 19-d study, 32 TGEV-seronegative piglets [Duroc × (Landrace ×Yorkshire)] with initial body weight of 7.63 ± 0.33 kg were weaned at 21 days of age and randomly divided into 4 treatments (*n* = 8): (1) Control group (piglets fed basal diet and infused with the sterile DMEM medium); (2) TGEV group (piglets fed basal diet and infused with TGEV); (3) TGEV + ATRA5 group (piglets fed basal diet and received oral administration of 5 mg ATRA daily and infused with TGEV); and (4) TGEV + ATRA15 group (piglets fed basal diet and received oral administration of 15 mg ATRA daily and infused with TGEV). Pigs were housed individually in metabolic cages (1.5 m × 0.7 m × 1.0 m) of two environmentally controlled rooms (25–28 °C) with strictly control to avoid cross-infection. The basal diet (Table 1) was formulated to meet or exceed the swine nutrient requirements recommended by the National Research Council (NRC, 2012) [17], except for vitamin A, which was not prepared in the vitamin premix. ATRA (≥ 98% HPLC) was purchased from Sigma-Aldrich (Shanghai, China) and dissolved in corn oil at a dose of 5 or 15 mg/mL. On d 14 of the trial, piglets in the challenged groups were orally administered TGEV at a dose of 2 × 10^8.85^ TCID50 (TGEV was provided by Prof. Zhiwen Xu, College of Veterinary Medicine, Sichuan Agricultural University), while those in control group received the same volume of sterile DMEM medium. The dose of TGEV was chosen according to our preliminary studies, which showed that it significantly induced diarrhea in weaned piglets. All piglets were executed on d 19 to collect samples.

**Table 1:** Composition and nutrient levels of the basal diet (as-fed basis)

Ingredients	Content, %	Nutrient level^3^	Content, %
Maize	30.80	Digestible energy, MJ/kg	14.73
Extruded maize	30.00	Crude protein	18.26
Soybean meal	9.00	Calcium	0.76
Extruded soybean	8.50	Total phosphorus	0.57
Soybean protein concentrate	5.20	Available phosphorus	0.39
Sucrose	2.00	Digestible Lysine	1.39
Soybean oil	1.70	Digestible Methionine	0.45
Fish meal	4.00	Digestible Threonine	0.78
Whey powder	6.00	Digestible Tryptophan	0.22
L-Lysine-HCl (78%)	0.45		
DL-Methionine (98%)	0.14		
L-Threonine (98%)	0.04		
L-Tryptophan (98%)	0.02		
CaCO_3_	0.75		
CaHPO_4_	0.50		
Choline chloride	0.15		
NaCl	0.20		
Benzoic acid	0.30		
Vitamin premix^1^	0.05		
Mineral premix^2^	0.20		
Total	100.00		

^1^Vitamin premix provided the following per kg of diets: Vitamin D_3_, 3,000 IU; Vitamin E, 60 IU; Vitamin K_3_, 4.0 mg; Vitamin B_1_, 4.0 mg; Vitamin B_2_, 8.0 mg; Vitamin B_6_, 6.0 mg; Vitamin B_12_, 0.06 mg; Biotin, 0.3 mg; Niacin, 50 mg; Pantothenic, 30 mg; Folic acid, 2.0 mg

^2^Mineral premix provided the following per kg of diets: Fe (FeSO_4_·H_2_O), 100 mg; Cu (CuSO_4_·5H_2_O), 120 mg; Mn (MnSO_4_·H_2_O), 20 mg; I (KI), 0.3 mg; Zn (ZnSO_4_·H_2_O), 100 mg; Se (Na_2_SeO_3_), 0.3 mg

^3^Nutrient levels were calculated values

### Growth performance and diarrhea rate

Piglets were weighed individually on d 0, 14 and 19 of the experiment, and the daily feed intake was recorded. These values were used to calculate average daily gain (ADG), average daily feed intake (ADFI) and feed to gain ratio (F/G). After the TGEV challenge, piglets were observed daily to record the health status and diarrhea incidence. Fecal consistency was scored as described by Pu et al. [18]: 0 = normal, 1 = pasty, 2 = semiliquid, and 3 = liquid. Score ≥ 2 was considered diarrhea. Diarrhea rate (%) = (days of piglet diarrhea/test days) × 100.

### Sample collection

On day 19 of the trial, blood samples were collected in 10-mL vacuum tubes without anticoagulant via anterior vein cava of each piglet, and stood at room temperature for 30 min. Blood was centrifuged at 3,500 r/min for 10 min at 4 °C, and serum samples were collected and stored at −20 °C until further assay. All piglets were euthanized by intramuscular injection of Shumianning (Active ingredients: tiletamine, xylazine and midazolam; 0.08 mL/kg body weight; Zhengzhou Huiji Animal Health Care Products Company, Henan, China). Then, the jejunum was quickly isolated and flushed with ice-cold saline. Mucosal samples from the middle portion of jejunum were scraped and collected by using a sterile glass microscope slide, quickly frozen in liquid nitrogen, and then stored at −80 °C until analysis.

### Disaccharidase activity measurement

Approximately 0.5 g of frozen jejunal mucosa was homogenized in ice-cold physiological saline at a ratio of 1:9 (w/v) for 15 min. The homogenate was then centrifuged at 3,500 r/min for 15 min at 4 °C, and the supernatant was collected for disaccharidase activity measurement. The activities of maltase, lactase and sucrase in the jejunal mucosa were measured by commercial kits (Nanjing Jiancheng Bioengineering Institute, Jiangsu, China) according to the manufacturer’s instructions. The concentration of total protein in the supernatant was determined by the BCA protein assay kit (Nanjing Jiancheng Bioengineering Institute, Jiangsu, China). The activities of disaccharidase were presented as U/mg protein.

### Determination of serum diamine oxidase activity

The activity of diamine oxidase (DAO) in serum was detected according to the instructions of a commercially available enzyme-linked immunosorbent assay (ELISA) kit (Jiangsu Meimian Biotechnology Co., Ltd., Jiangsu, China).

### Determination of cytokines and secretory immunoglobulin A concentrations in jejunal mucosa

The concentration of cytokines (IL-1β, IL-6, IL-8, IL-10 and TNF-α) and secretory immunoglobulin A (sIgA) in jejunal mucosa of piglets were measured by using ELISA kits (Jiangsu Meimian Biotechnology Co., Ltd., Jiangsu, China) specific for swine according to the manufacturer’s instructions. Before assays, about 0.5 g of frozen jejunal mucosa was homogenized in ice-cold saline and prepared into a 10% homogenate, and then centrifuged (3,500 r/min, 15 min, 4 °C). The collected supernatant was used to determine cytokines and sIgA concentration.

### Quantitative real-time PCR analysis

Total RNA from the jejunal mucosa was extracted using TRIzol reagent (TaKaRa Biotechnology Co., Ltd., Dalian, China) in accordance with the manufacturer’s instructions. The concentration and purity of total RNA were analyzed by using a spectrophotometer (Beckman Coulter DU800; Beckman Coulter Inc.). 1 µg of total RNA was reverse transcribed into cDNA using the PrimeScripte RT reagent kit (TaKaRa). Then, the synthesized cDNA was diluted (1:4) and quantitative real-time PCR was performed using SYBR Premix Ex Taq™ reagents (TaKaRa) and CFX96 Real-Time PCR Detection System (Bio-Rad, Hercules, CA, USA). The primer sequences were synthesized by TaKaRa Biotechnology Co., Ltd. (Dalian, China) and listed in Table 2. The relative mRNA expression of target genes was calculated with the 2^−ΔΔCt^ method and using *β-actin* as the reference gene [19].

**Table 2:** Primer sequences of the target genes

Gene	Primer sequence (5´→3´)	Product length, bp	GenBank accession
*SGLT1*	F: GCAACAGCAAAGAGGAGCGTAT	137	NM_001164021.1
	R: GCCACAAAACAGGTCATAGGTC		
*GLUT2*	F: GACACGTTTTGGGTGTTCCG	149	NM_001097417.1
	R: GAGGCTAGCAGATGCCGTAG		
*PepT1*	F: GCCAAAGTCGTCAAGTGC	100	NM_214347
	R: GGTCAAACAAAGCCCAGA		
*SLC7A1*	F: TCTTTGCAGGTCGTTTGGGA	137	NM_001012613.1
	R: GGCTGATCACCTGTTGGAGT		
*TNF-α*	F: CGTGAAGCTGAAAGACAACCAG R: GATGGTGTGAGTGAGGAAAACG	121	NM_214022.1
*IL-1β*	F: CAGCTGCAAATCTCTCACCA R: TCTTCATCGGCTTCTCCACT	112	NM_214055.1
*IL-6*	F: TTCACCTCTCCGGACAAAAC R: TCTGCCAGTACCTCCTTGCT	122	NM_001252429.1
*IL-8*	F: AGTTTTCCTGCTTTCTGCAGCT R: TGGCATCGAAGTTCTGCACT	72	NM_213867.1
*IL-10*	F: CCTGGAAGACGTAATGCCGA R: CACGGCCTTGCTCTTGTTTT	148	NM_214041.1
*RIG-I*	F: AGAGCAGCGGCGGAATC	82	NM_213804.2
	R: GGCCATGTAGCTCAGGATGAA		
*MDA5*	F: TCCGGGAAACAGGCAACTC	75	NM_001100194.1
	R: CAAAGGATGGAGAGGGCAAGT		
*MAVS*	F: TGGGTACAGTCCTTCATCGG R: GGGTAACTTGGCTCATCCTCT	116	NM_020746.5
*TLR2*	F: TCACTTGTCTAACTTATCATCCTCTTG	162	XM_005653576.3
	R: TCAGCGAAGGTGTCATTATTGC		
*TLR3*	F: TGGAAAAAGGAATGGCCAGC R: ACAAGGCAAACTCCTGCTCA	265	NM_001042467.3
*TLR4*	F: TTACAGAAGCTGGTTGCCGT R: TCCAGGTTGGGCAGGTTAGA	152	NM-001293316.1
*TLR7*	F: CAATGGTCCCTGAGCGTTTG R: AGCCTGGTTGAAGACAGCAG	126	NM_016562
*TLR9*	F: CACGACAGCCGAATAGCAC	121	NM_213958.1
	R: GGGAACAGGGAGCAGAGC		
*MyD88*	F: GTGCCGTCGGATGGTAGTG	65	NM001099923
	R: TCTGGAAGTCACATTCCTTGCTT		
*TRIF*	F: CAAGTGGAGGAAGGAACAGG R: CAACTGCGTCTGGTAGGACA	139	XM_003362039.1
*TRAF6*	F: CAAGAGAATACCCAGTCGCACA R: ATCCGAGACAAAGGGGAAGAA	122	NM-001105286.1
*β-actin*	F: GGATGACGATATTGCTGCGC R: GATGCCTCTCTTGCTCTGGG	190	XM_003124280.5

*SGLT1* Sodium-glucose cotransporter 1, *GLUT2* Glucose transporter type 2, *PepT1* Oligopeptide transporter 1, *SLC7A1* Solute carrier family 7, *TNF-α* Tumor necrosis factor-α, *IL* Interleukin, *RIG-I* Retinoic acid-inducible gene I, *MDA5* Melanoma differentiation associated gene 5, *MAVS* Mitochondrial antiviral signaling protein, *TLR* Toll-like receptor, *MyD88* Myeloid differentiation factor 88, *TRIF* TIR domain-containing adaptor inducing interferon β, *TRAF6* TNF receptor-associated factor 6

### Western blot analysis

Proteins from jejunal mucosa were extracted with RIPA lysis buffer (Beyotime Biotechnology, Shanghai, China), and the protein concentrations in the supernatants were measured by a BCA protein assay kit (Thermo Scientific, Waltham, MA, USA). Equal amounts of protein (25 µg) were resolved by sodium dodecyl sulphate–polyacrylamide gel electrophoresis (SDS-PAGE) and transferred to polyvinylidene difluoride (PVDF) membranes using a wet Trans-Blot system (Bio-Rad). The membranes were blocked with 5% nonfat dry milk for 1 h at room temperature, and then incubated overnight at 4 °C with corresponding primary antibodies: anti-ZO-1 (Invitrogen, USA), anti-occludin (Abcam, UK), anti-claudin-1(Abcam, UK), anti-IκBα (Cell Signaling Technology, Danvers, MA, USA), anti-NF-κB p65 (Cell Signaling Technology), anti-p-NF-κB p65 (Cell Signaling Technology) and anti-β-actin (Santa Cruz, USA). After washing, the blots were incubated with goat anti-rabbit/mouse IgG-HRP secondary antibody (Santa Cruz, USA) for 1 h at room temperature. Visualization of blots was conducted with the ECL chemiluminescence kit (Beyotime Biotechnology, Shanghai, China) and the ChemiDoc™ XRS Imager System (Bio-Rad). The protein bands were analyzed by Image Lab 5.1 software. The results were represented as the ratio of the optical density of the target protein bands to the respective β-actin band.

### Statistical analysis

The Shapiro–Wilk test was performed before statistical analysis to determine normality of variance. Data were analyzed by one-way analysis of variance (ANOVA) of SPSS 22.0 software (SPSS Inc., Chicago, IL, USA), using each piglet as the experimental unit. Differences between means were determined using Tukey’s multiple-comparison test. Moreover, the Chi-square test was used to analyze diarrhea rate. Results were presented as mean ± standard error (SE). *P* ≤ 0.05 was considered statistically significant.

## Results

### Growth performance and diarrhea

The results of growth performance and diarrhea were shown in Table 3. Compared with the control group, TGEV group significantly decreased ADFI and ADG, and increased F/G of piglets (*P* < 0.05). However, compared with TGEV group, 5 mg/d ATRA feeding significantly increased ADFI and ADG, and decreased F/G of piglets post-challenged (*P* < 0.05), while 15 mg/d ATRA feeding significantly increased ADG and decreased F/G of piglets post-challenged (*P* < 0.05). Furthermore, the diarrhea rate of piglets in TGEV group was significantly higher than that in the control group (*P* < 0.05). However, 5 and 15 mg/d ATRA feeding significantly alleviated the diarrhea of piglets induced by TGEV (*P* < 0.05).

**Table 3:** Effects of ATRA on growth performance and diarrhea of piglets challenged with TGEV

Items	Control	TGEV	TGEV + ATRA5	TGEV + ATRA15	*P-*value
Initial BW, kg	7.63 ± 0.12	7.62 ± 0.12	7.64 ± 0.12	7.63 ± 0.13	0.993
d 14 BW, kg	11.34 ± 0.34	11.37 ± 0.32	12.01 ± 0.28	11.39 ± 0.28	0.367
d 19 BW, kg	13.40 ± 0.38^ab^	12.88 ± 0.39^a^	14.26 ± 0.36^b^	13.32 ± 0.32^ab^	0.077
d 14–19 (Post-challenge)					
ADFI, g	669.20 ± 33.30^b^	563.00 ± 54.45^a^	711.33 ± 38.47^b^	647.15 ± 13.01^ab^	0.046
ADG, g	411.25 ± 18.46^b^	300.00 ± 47.96^a^	448.75 ± 24.53^b^	386.25 ± 19.72^b^	0.012
F/G	1.64 ± 0.09^a^	2.06 ± 0.20^b^	1.59 ± 0.03^a^	1.70 ± 0.07^a^	0.033
Diarrhea rate, %	0.00^a^	35.00^b^	5.00^a^	5.00^a^	< 0.001

*BW* Body weight, *ADFI* Average daily feed intake, *ADG* Average daily gain, *F/G* Feed to gain ratio. Data are presented as mean ± SE. ^a,b^Values in the same row with different superscripts letters are significantly different (*P* < 0.05)

### Intestinal digestion and absorption

**Fig. 1:**
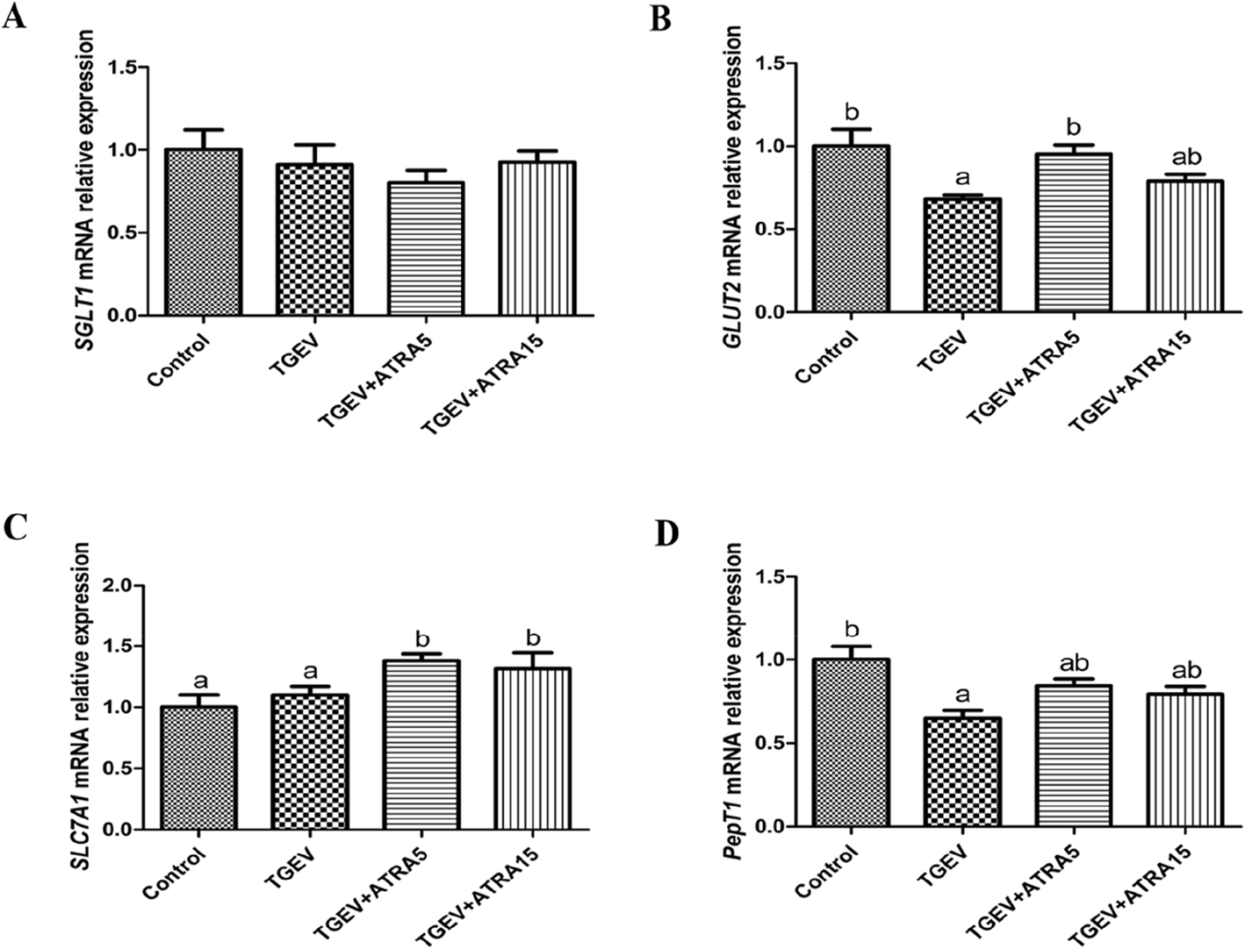
Effects of ATRA on the expressions of nutrient transporter-related genes in jejunal mucosa of piglets challenged with TGEV. The mRNA expressions of *SGLT1* (**A**), *GLUT2* (**B**), *SLC7A1* (**C**) and *PepT1* (**D**) in jejunal mucosa of piglets were analyzed by real-time PCR. Data are presented as mean ± SE. ^a,b^Values in the same row with different superscripts letters are significantly different (*P* < 0.05)

As shown in Table 4, the activity of sucrase in jejunal mucosa of piglets in TGEV group was lower than that in the control group (*P* < 0.05). However, 5 mg/d ATRA feeding significantly alleviated the decrease of sucrase activity induced by TGEV (*P* < 0.05). Nutrient transporter related genes expression in jejunal mucosa were shown in Fig. 1. The mRNA expressions of *GLUT2* and *PepT1* were significantly down-regulated in jejunal mucosa of piglets in TGEV group compared with the control group (*P* < 0.05). However, compared with TGEV group, 5 mg/d ATRA feeding significantly up-regulated the mRNA expressions of *GLUT2* and *SLC7A1* in jejunal mucosa of piglets (*P* < 0.05), while 15 mg/d ATRA feeding significantly up-regulated the mRNA expression of *SLC7A1* in jejunal mucosa of piglets (*P* < 0.05).

**Table 4:** Effects of ATRA on disaccharidase activity in jejunal mucosa of piglets challenged with TGEV

Items	Control	TGEV	TGEV + ATRA5	TGEV + ATRA15	*P*-value
Maltase, U/mg prot	261.15 ± 35.69	222.21 ± 24.42	256.97 ± 12.50	227.55 ± 28.04	0.643
Lactase, U/mg prot	96.36 ± 14.51	61.55 ± 10.18	97.56 ± 15.52	65.64 ± 10.00	0.101
Sucrase, U/mg prot	87.76 ± 9.37^b^	45.49 ± 6.94^a^	84.49 ± 9.78^b^	62.14 ± 12.93^ab^	0.018

Data are presented as mean ± SE. ^a,b^Values in the same row with different superscripts letters are significantly different (*P* < 0.05)

### Intestinal barrier integrity

**Fig. 2:**
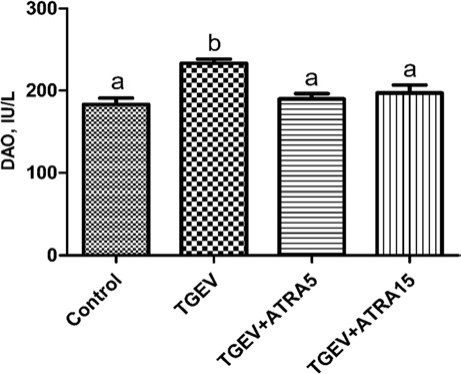
Effect of ATRA on serum DAO activity of piglets challenged with TGEV. The activity of diamine oxidase (DAO) in serum of piglets was detected by ELISA. Data are presented as mean ± SE. ^a,b^Values in the same row with different superscripts letters are significantly different (*P* < 0.05)

**Fig. 3:**
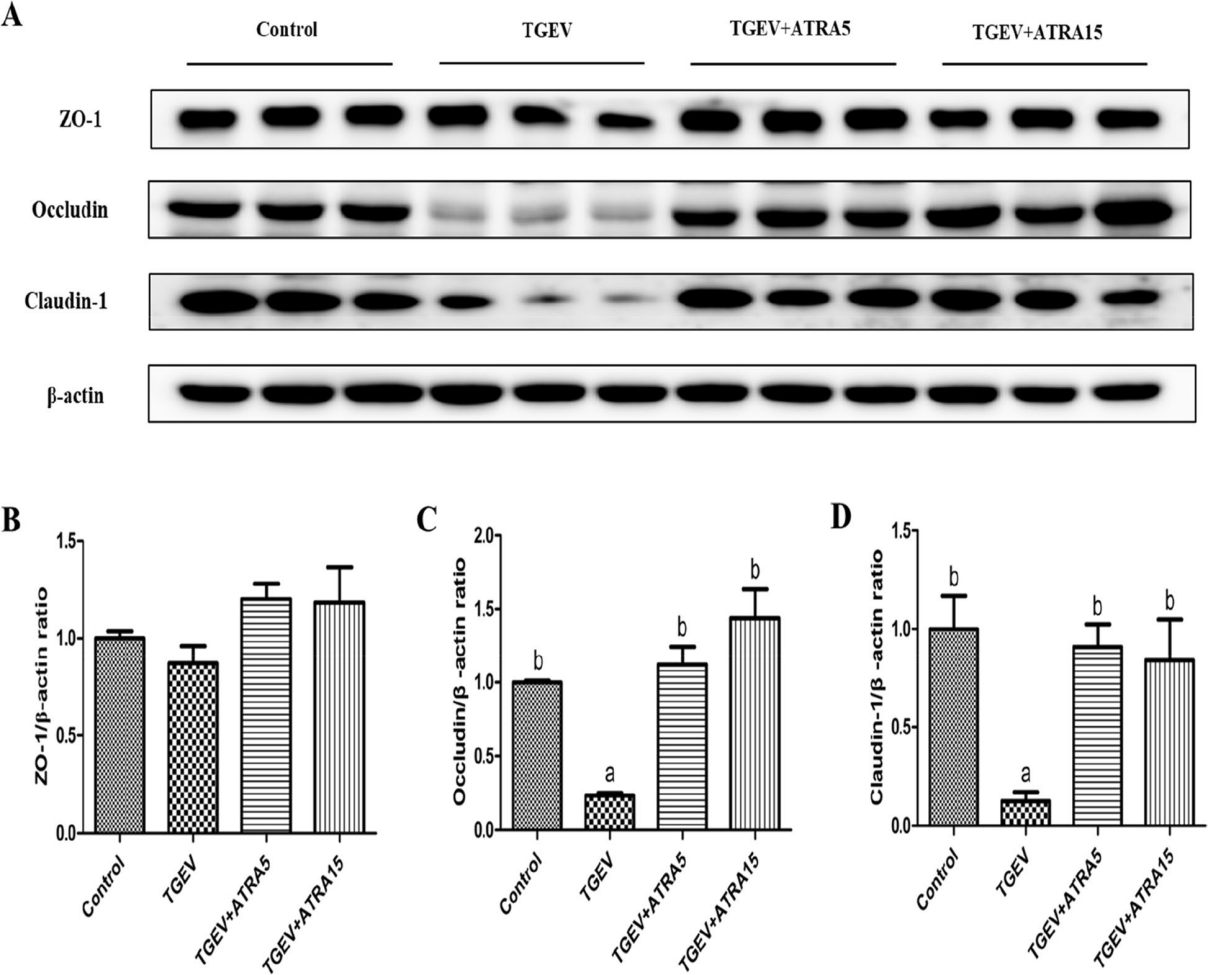
Effects of ATRA on the tight junction protein levels in jejunal mucosa of piglets challenged with TGEV. **A–D** The protein levels of ZO-1, occludin and claudin-1 in jejunal mucosa of piglets were analyzed by Western blot. Data are presented as mean ± SE. ^a,b^Values in the same row with different superscripts letters are significantly different (*P* < 0.05)

DAO release is an important marker for assessing intestinal permeability. To determine the effects of ATRA on intestinal barrier integrity of weaned piglets challenged with TGEV, we measured the activity of DAO in serum. As shown in Fig. 2, the activity of DAO in serum of piglets in TGEV group was higher than that in the control group (*P* < 0.05). However, 5 and 15 mg/d ATRA feeding significantly inhibited the increase of DAO activity induced by TGEV (*P* < 0.05). To further identify whether ATRA alleviated TGEV-induced intestinal barrier integrity damage of piglets, the levels of tight junction protein in jejunal mucosa were detected (Fig. 3). Compared with the control group, TGEV group significantly decreased the protein levels of occludin and claudin-1 in jejunal mucosa of piglets (*P* < 0.05). However, 5 and 15 mg/d ATRA feeding significantly alleviated the TGEV-induced decrease of occludin and claudin-1 protein levels (*P* < 0.05).

### Intestinal inflammation

**Fig. 4:**
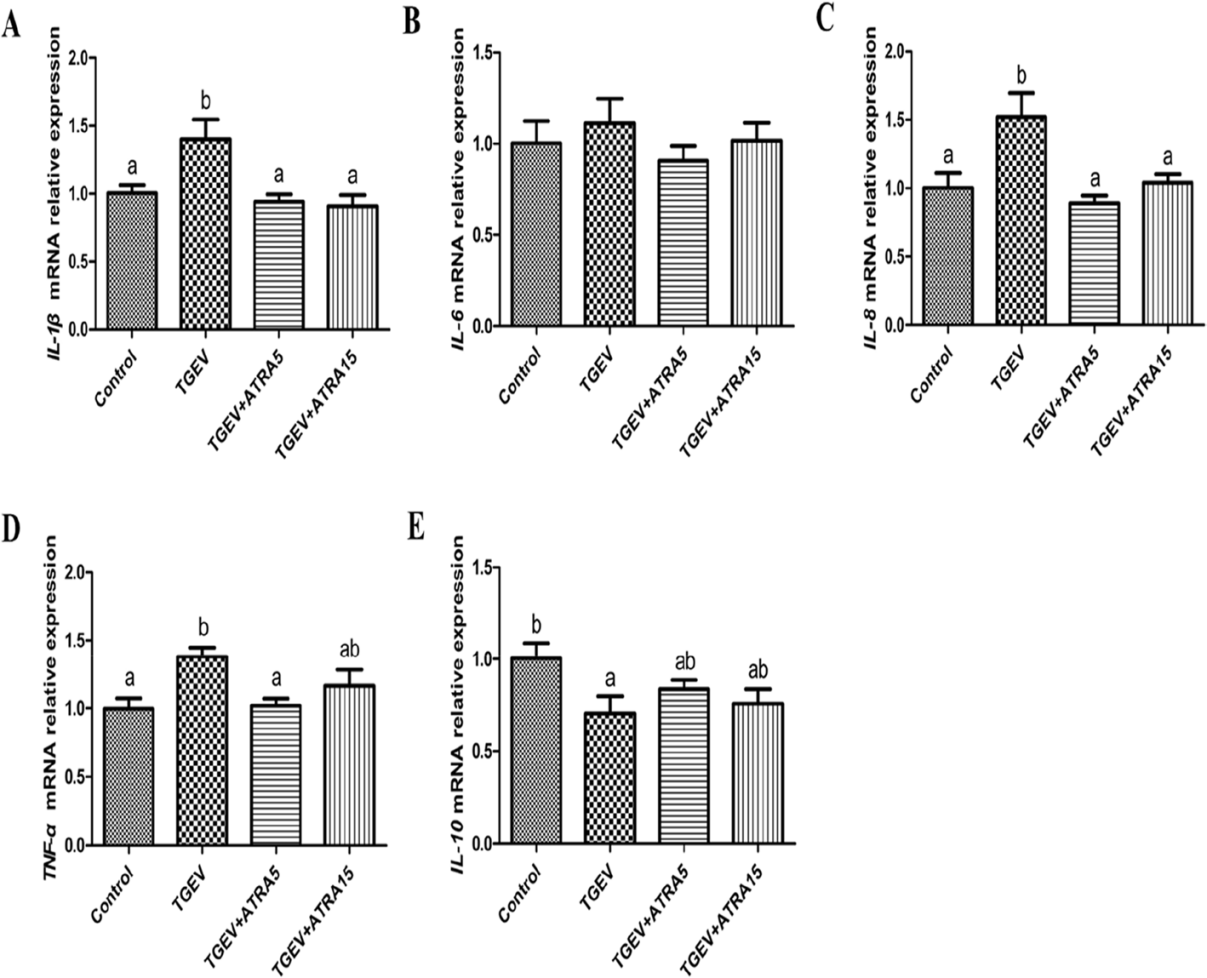
Effects of ATRA on the mRNA expressions of inflammatory cytokines in jejunal mucosa of piglets challenged with TGEV. The mRNA expressions of *IL-1β* (**A**), *IL-6* (**B**), *IL‐8* (**C**), *TNF‐α* (**D**) and *IL-10* (**E**) in jejunal mucosa of piglets were analyzed by real‐time PCR. Data are presented as mean ± SE. ^a,b^Values in the same row with different superscripts letters are significantly different (*P* < 0.05)

To evaluate whether ATRA can alleviate TGEV-induced intestinal inflammatory response of piglets, the mRNA expressions of inflammatory cytokines in jejunal mucosa were analyzed by real-time PCR. As shown in Fig. 4, TGEV group significantly upregulated the mRNA abundance of *IL-1β*, *IL-8* and *TNF-α*, and downregulated *IL-10* mRNA expression in jejunal mucosa of piglets compared with the control group (*P* < 0.05). However, 5 and 15 mg/d ATRA feeding significantly inhibited the upregulation of *IL-1β* and *IL-8* mRNA expressions induced by TGEV (*P* < 0.05). Meanwhile, 5 mg/d ATRA feeding significantly alleviated the upregulation of *TNF-α* mRNA abundance induced by TGEV (*P* < 0.05). To further determine the effects of ATRA on intestinal inflammatory response of weaned piglets challenged with TGEV, we detected the concentrations of cytokines and sIgA in jejunal mucosa. As shown in Table 5, TGEV group significantly increased IL-1β and IL-8 concentrations, and decreased IL-10 and sIgA concentrations in jejunal mucosa of piglets compared with the control group (*P* < 0.05). However, 5 mg/d ATRA feeding significantly inhibited the elevation of IL-1β and IL-8 concentrations and the reduction of IL-10 and sIgA concentrations induced by TGEV (*P* < 0.05). Furthermore, 15 mg/d ATRA feeding significantly alleviated the increase of IL-8 concentration and the decrease of IL-10 concentration induced by TGEV (*P* < 0.05).

**Table 5:** Effects of ATRA on the concentrations of cytokines and sIgA in jejunal mucosa of piglets challenged with TGEV

Items	Control	TGEV	TGEV + ATRA5	TGEV + ATRA15	*P*-value
IL-1β, pg/mg prot	12.86 ± 0.92^a^	16.53 ± 0.70^b^	12.37 ± 1.35^a^	14.62 ± 0.62^ab^	0.017
IL-6, pg/mg prot	196.40 ± 20.35	220.65 ± 9.08	178.30 ± 21.87	203.46 ± 12.86	0.357
IL-8, pg/mg prot	112.38 ± 6.82^a^	152.20 ± 6.52^b^	114.89 ± 10.65^a^	118.93 ± 5.75^a^	0.003
IL-10, pg/mg prot	36.90 ± 3.16^b^	23.70 ± 2.12^a^	33.71 ± 2.58^b^	34.78 ± 2.18^b^	0.005
TNF-α, pg/mg prot	93.51 ± 7.62	103.41 ± 5.54	84.75 ± 7.47	96.64 ± 5.06	0.261
sIgA, µg/mg prot	19.17 ± 1.08^b^	13.77 ± 0.84^a^	18.51 ± 1.41^b^	18.10 ± 1.32^ab^	0.013

*IL* Interleukin, *TNF-α* Tumor necrosis factor-α, *sIgA* Secretion immunoglobulin A. Data are presented as mean ± SE. ^a,b^Values in the same row with different superscripts letters are significantly different (*P* < 0.05)

### NF-κB signaling pathway related protein expressions in jejunal mucosa

**Fig. 5:**
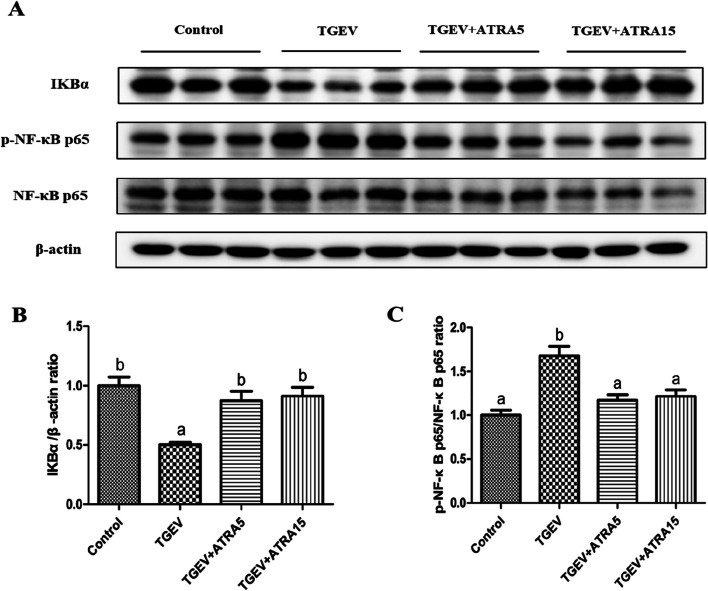
Effects of ATRA on the levels of NF-κB signaling pathway related proteins in jejunal mucosa of piglets challenged with TGEV. **A–C** The protein levels of IκBα and p-NF-κB p65 in jejunal mucosa of piglets were analyzed by Western blot. Data are presented as mean ± SE. ^a,b^Values in the same row with different superscripts letters are significantly different (*P* < 0.05)

As shown in Fig. 5, compared with the control group, TGEV group significantly decreased the protein level of IκBα, and increased the phosphorylation level of NF-κB p65 in jejunal mucosa of piglets (*P* < 0.05). However, 5 and 15 mg/d ATRA feeding significantly inhibited the downregulation of IκBα protein level and the upregulation of NF-κB p65 phosphorylation level induced by TGEV (*P* < 0.05).

### TLRs/RLRs signaling pathway related gene expressions in jejunal mucosa

**Fig. 6:**
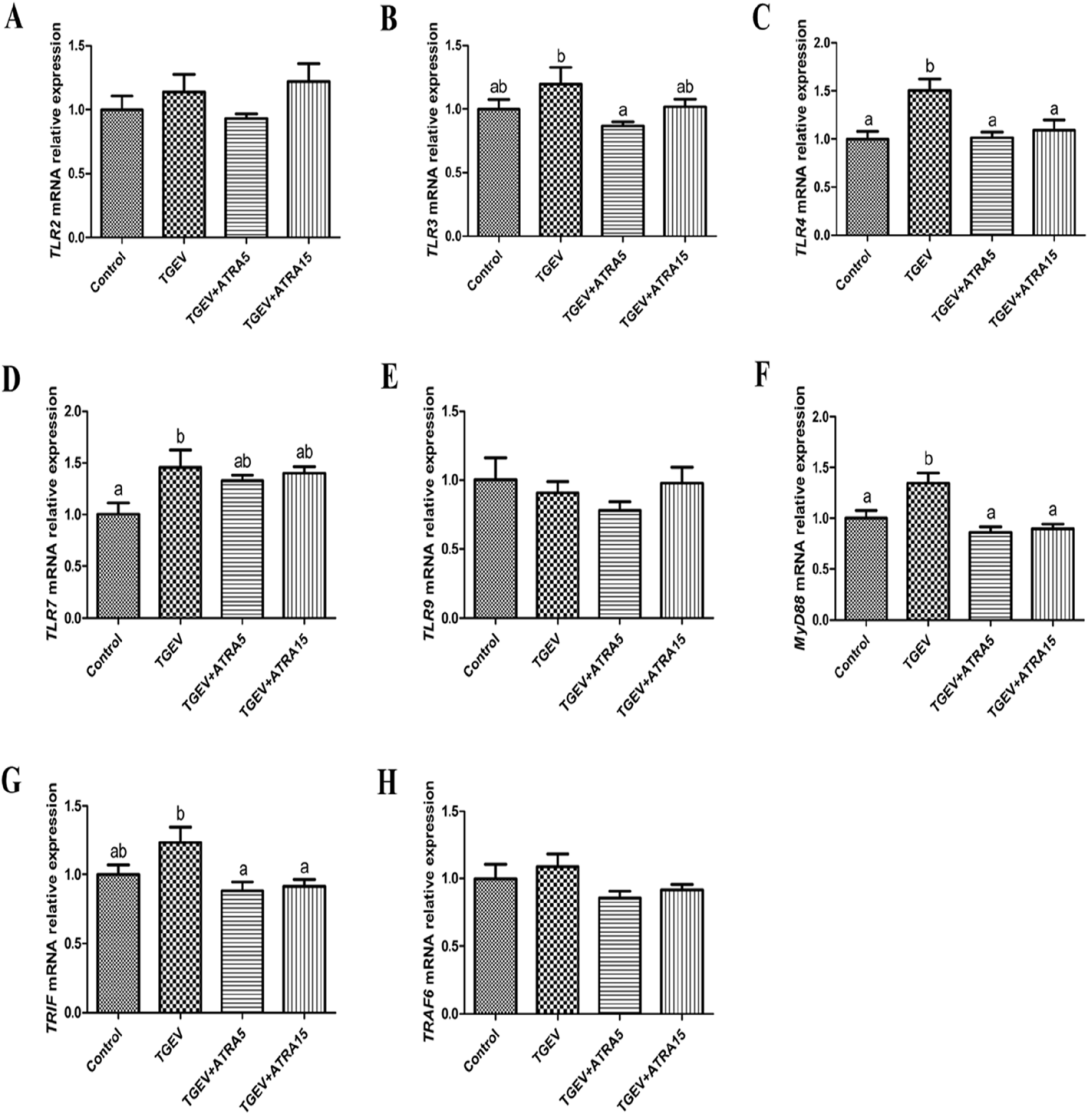
Effects of ATRA on the expressions of TLRs signaling pathway related genes in jejunal mucosa of piglets challenged with TGEV. The mRNA expressions of *TLR2* (**A**), *TLR3* (**B**), *TLR4* (**C**), *TLR7* (**D**), *TLR9* (**E**), *MyD88* (**F**), *TRIF* (**G**) and *TRAF6* (**H**) in jejunal mucosa of piglets were analyzed by real-time PCR. Data are presented as mean ± SE. ^a,b^Values in the same row with different superscripts letters are significantly different (*P* < 0.05)

**Fig. 7:**
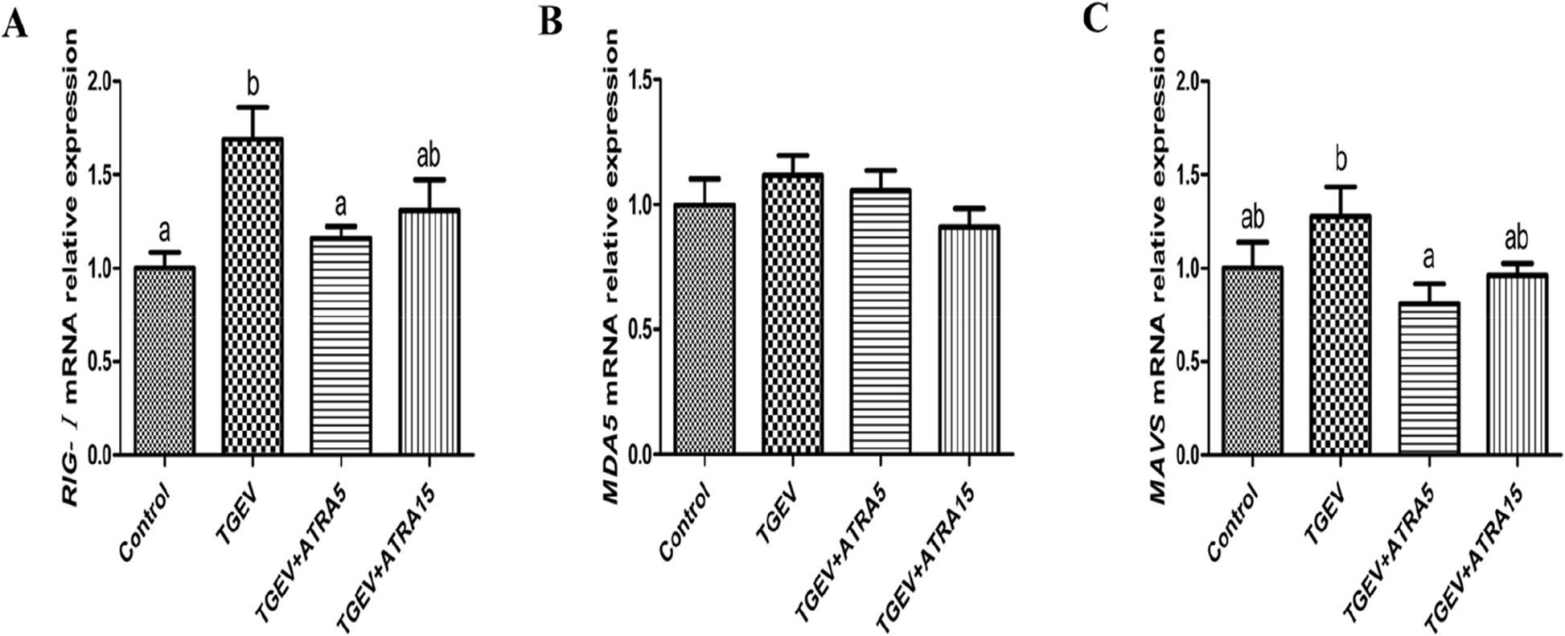
Effects of ATRA on the expressions of RLRs signaling pathway related genes in jejunal mucosa of piglets challenged with TGEV. The mRNA expressions of *RIG-I* (**A**), *MDA5* (**B**) and *MAVS* (**C**) in jejunal mucosa of piglets were analyzed by real-time PCR. Data are presented as mean ± SE. ^a,b^Values in the same row with different superscripts letters are significantly different (*P* < 0.05)

As shown in Fig. 6, the mRNA expressions of *TLR4*, *TLR7* and *MyD88* were significantly increased in jejunal mucosa of piglets in TGEV group compared with the control group (*P* < 0.05). However, 5 and 15 mg/d ATRA feeding significantly inhibited the upregulation of *TLR4*, *MyD88* and *TRIF* mRNA expressions induced by TGEV (*P* < 0.05). Meanwhile, 5 mg/d ATRA feeding significantly suppressed the upregulation of *TLR3* mRNA expression induced by TGEV (*P* < 0.05). Furthermore, the mRNA expression of *RIG-I* was significantly upregulated in jejunal mucosa of piglets in TGEV group compared with the control group, and 5 mg/d ATRA feeding significantly inhibited the upregulation of *RIG-I* and *MAVS* mRNA expressions induced by TGEV (*P* < 0.05, Fig. 7).

## Discussion

Piglet diarrhea has always been an important bottleneck restricting the healthy development of the global pig industry. Porcine transmissible gastroenteritis virus (TGEV) is one of the main pathogens causing diarrhea and death of piglets, which brings serious economic losses to the pig industry [20]. All-*trans* retinoic acid is the main active metabolite of vitamin A, and plays an important role in regulating reproduction, vision, cell growth and differentiation, immune response and apoptosis [21, 22]. In recent years, studies have shown that ATRA has broad-spectrum antiviral properties against a number of pathogenic human and animal viruses, such as enterovirus 71 [23], hepatitis C virus [24] and norovirus [25]. However, it is unclear whether ATRA can alleviate TGEV infection in piglets. Previous studies have reported that TGEV infection reduced growth performance and resulted in severe diarrhea in piglets [26, 27]. Consistent with previous reports, our current studies found that TEGV challenge significantly decreased ADFI and ADG, increased F/G, and caused severe diarrhea of piglets. However, 5 and 15 mg/d ATRA feeding significantly alleviated the growth inhibition and diarrhea of piglets induced by TGEV challenge, indicating that ATRA can alleviate the symptoms of TGEV infection in piglets. This may be associated with the changes in the intestinal barrier integrity.

Intestine is not only the major site for digestion and absorption of nutrients, but also an important barrier to resist the invasion of pathogenic bacteria. Previous studies have shown that the porcine small intestine is the main target organ of TGEV infection [28]. TGEV replicates in enterocytes covering the villi of the porcine small intestine, causing villus atrophy and crypt hyperplasia, and impairing intestinal barrier integrity, which will disturb the absorption of nutrients, and then lead to diarrhea and growth retardation in piglets [5]. Therefore, we speculate that ATRA may alleviate the growth retardation and diarrhea of piglets induced by TGEV infection by improving the intestinal barrier function. DAO is known to be an intracellular enzyme mainly synthesized by mammalian intestinal epithelial cells, which is released into the blood when the intestinal barrier is damaged, so the activity of DAO in serum can indirectly reflect the intestinal permeability [29]. In the present study, TGEV challenge significantly increased serum DAO activity of piglets, whereas 5 and 15 mg/d ATRA feeding significantly inhibited the increase of DAO activity induced by TGEV. These results suggested that ATRA could alleviate the TGEV-induced intestinal barrier damage of piglets. Tight junction proteins [(occludin, claudin and intracellular plaque proteins (ZO and cingulin)] are the constituents of tight junctions and important regulators of paracellular permeability, and play an important role in regulating the integrity of intestinal barrier [30]. Previous studies reported that TGEV infection induced IPEC-J2 cells damage by down-regulating the expression of tight junction protein (ZO-1 and occludin) and adhesion junction protein (E-cadherin) [8]. To further identify the protective effect of ATRA on the TGEV-induced intestinal barrier integrity damage of piglets, we detected the expressions of tight junction related proteins. Our results found that 5 and 15 mg/d ATRA feeding significantly alleviated the TGEV-induced decrease of occludin and claudin-1 protein levels in jejunal mucosa of piglets. Similar results also reported that ATRA treatment significantly alleviated LPS-induced downregulation of ZO-1, occludin and claudin-1 protein levels in IPEC-J2 cells [31]. These results indicated that ATRA could alleviate the TGEV-induced intestinal barrier damage of piglets by promoting the expression of intestinal tight junction proteins. In addition, our study found that 5 mg/d ATRA feeding significantly increased the sucrase activity and the mRNA expressions of *GLUT2* and *SLC7A1* in jejunal mucosa of piglets infected with TGEV. Sucrase is a kind of disaccharidase located on the brush border of the small intestine. GLUT2 and SLC7A1 are the transport carriers of glucose and amino acid in small intestinal mucosa, which are important indicators to judge the intestinal digestion and absorption ability for nutrient [32]. The above results indicated that ATRA can promote the digestion and absorption of nutrients by alleviating the intestinal barrier damage induced by TGEV challenge, thus improving the growth performance and inhibiting diarrhea of piglets.

Virus invasion always leads to inflammatory reaction, which is the key medium for the host to resist microbial pathogens. However, excessive production of proinflammatory cytokines will lead to tissue damage [33]. Previous studies showed that TGEV infection significantly upregulated the mRNA levels of proinflammatory cytokines (*IL-1β*, *IL-6*, *IL-8* and *TNF-α*) in ST cells and IPEC-J2 cells [7]. Xia et al. [5] reported that TGEV infection significantly decreased the number of sIgA positive cells and dendritic cells (DCs), and enhanced the mRNA expression levels of proinflammatory cytokines *IL-1β*, *IL-6* and *TNF-α* in the jejunum of piglets. Our current study also found that TGEV challenge significantly upregulated the mRNA abundance of proinflammatory cytokines *IL-1β*, *IL-8* and *TNF-α*, and downregulated anti-inflammatory cytokines *IL-10* mRNA expression in jejunal mucosa of piglets, which was consistent with the results of TGEV-induced intestinal barrier damage in piglets. These results indicated that the pathogenesis of TGEV is closely related to the intestinal inflammatory response. As an immunomodulator, ATRA plays important roles in regulating immune response and anti-inflammation [9]. Xu et al. [34] reported that ATRA treatment significantly inhibited the production of proinflammatory cytokines IL-1β, IL-6, IL-17 and TNF-α induced by LPS in bovine adipocytes. Sierra et al. [35] found that ATRA can ameliorate the inflammatory response during initiation of diabetic nephropathy by inhibiting the expressions of proinflammatory factors (*IL-1 β*, *IL-6*, *IL-13*, *IL-2* and *TNF-α*). However, it is unclear whether ATRA can attenuate the intestinal inflammatory response induced by TGEV. Similar to previous reports, our current studies found that ATRA feeding alleviated TGEV-induced intestinal inflammatory response in piglets by inhibiting the expressions of proinflammatory factors (*IL-1β*, *IL-8* and *TNF-α*). To further verify the protective effect of ATRA on the TGEV‐induced intestinal inflammatory response in piglets, we detected the concentrations of cytokines and sIgA in jejunal mucosa. Our study found that 5 mg/d ATRA feeding significantly inhibited the elevation of IL-1β and IL-8 concentrations and the reduction of IL-10 and sIgA concentrations induced by TGEV, which was consistent with the results that ATRA could alleviate the intestinal barrier damage induced by TGEV in piglets. These results indicated that ATRA can alleviate TGEV-induced intestinal barrier damage in piglets by inhibiting inflammatory response.

To further study the mechanism by which ATRA alleviates TGEV-induced intestinal inflammatory response in piglets, we investigated the role of Toll-like receptors (TLRs) and RIG-I like receptors (RLRs) signaling pathways. Studies have shown that TLRs and RLRs are two main pattern recognition receptors (PRRs) for detecting virus pathogen-associated molecular patterns (PAMPs), which play a key role in regulating innate immune response [36]. TLRs are cell membrane pattern recognition receptors, including 13 members, such as TLR2, TLR3, TLR4, TLR7 and TLR9, which recognize viral glycoprotein, double-stranded RNA, single-stranded RNA or DNA [37]. RLRs, including RIG-I, MDA5 and LGP2, are cytoplasmic proteins that recognize viral RNA [38]. After the virus invades the host cell, the PRRs in the host cell will recognize the virus PAMPs, recruit specific intracellular adaptor proteins (MyD88, TRIF or MAVS), and activate transcription factors (NF-κB, AP-1 and IRF3) through a series of signal transduction, thus regulating the production of inflammatory cytokines and interferon [39]. Cao et al. [40] found that porcine epidemic diarrhea virus infection can induce NF-κB activation through the TLR2, TLR3 and TLR9 signaling pathways, thus inducing inflammatory response in porcine intestinal epithelial cells. Ding et al. [41] found that TGEV infection can induce inflammatory response in PK-15 cells by activating RLRs-mediated NF-κB signaling pathway. Similar to previous reports, our current studies found that TGEV challenge significantly upregulated the mRNA expressions of *TLR4*, *TLR7*, *RIG-I* and *MyD88* as well as the phosphorylation level of NF-κB p65, and decreased the protein level of IκBα in jejunal mucosa of piglets. These results indicated that TGEV may induce intestinal inflammation in piglets by activating NF-κB signaling pathway mediated by TLR4, TLR7 and RIG-I. Previous studies have reported that ATRA can attenuate lipopolysaccharide-induced inflammatory responses by suppressing TLR4/NF-κB signaling pathway in rat mammary tissue [11]. However, it is unclear whether ATRA can alleviate TGEV-induced intestinal inflammatory response in piglets by inhibiting the TLRs and RLRs mediated NF-κB signaling pathway. Our study found that 5 mg/d ATRA feeding significantly downregulated the mRNA expressions of *TLR3*, *TLR4*, *RIG-I* and their downstream signaling molecules (*MyD88*, *TRIF* and *MAVS*) as well as the phosphorylation level of NF-κB p65, and increased the protein level of IκBα in jejunal mucosa of piglets infected with TGEV. This was consistent with the results that ATRA could alleviate TGEV-induced intestinal inflammatory response in piglets. The above results suggested that ATRA can alleviate TGEV-induced intestinal inflammatory response in piglets by inhibiting the TLR3, TLR4 and RIG-I mediated NF-κB signaling pathway.

## Conclusions

In conclusion, our results indicated that ATRA alleviated TGEV-induced intestinal barrier damage by inhibiting inflammatory response, thus improving the growth performance and inhibiting diarrhea of piglets. The mechanism was associated with the inhibition of NF-κB signaling pathway mediated by TLR3, TLR4 and RIG-I. This provides a new strategy for the treatment of TGEV infection.

## Data Availability

The datasets used during the current study are available from the corresponding author on reasonable request.

## Abbreviations

ADFI: Average daily feed intake
ADG: Average daily gain
ATRA: All-*trans* retinoic acid
DAO: Diamine oxidase
F/G: The ratio of feed to gain
GLUT2: Glucose transport protein 2
IκBα: Inhibitor kappa B alpha
IL: Interleukin
MAVS: Mitochondrial antiviral signaling protein
MDA5: Melanoma differentiation associated gene 5
MyD88: Myeloid differentiation factor 88
NF-κB: Nuclear factor-κB
PepT1: Oligopeptide transporter 1
RIG-I: Retinoic acid-inducible gene-I
SGLT1: Sodium/glucose co-transporter 1
sIgA: Secretory immunoglobulin A
SLC7A1: Solute carrier family 7
TGEV: Transmissible gastroenteritis virus
TLR: Toll-like receptor
TNF-α: Tumor necrosis factor-α
TRAF6: TNF receptor-associated factor 6
TRIF: TIR domain-containing adaptor inducing interferonβ
ZO-1: Zonula occludens-1
